# A Trigger for Opioid Misuse: Chronic Pain and Stress Dysregulate the Mesolimbic Pathway and Kappa Opioid System

**DOI:** 10.3389/fnins.2016.00480

**Published:** 2016-11-07

**Authors:** Nicolas Massaly, Jose A. Morón, Ream Al-Hasani

**Affiliations:** ^1^Basic Research Division, Department of Anesthesiology, Washington University School of MedicineSt. Louis, MO, USA; ^2^Washington University Pain Center, Department of Anesthesiology, Washington University School of MedicineSt. Louis, MO, USA

**Keywords:** kappa opioid receptor, dopamine, chronic pain, reward, stress, psychological

## Abstract

Pain and stress are protective mechanisms essential in avoiding harmful or threatening stimuli and ensuring survival. Despite these beneficial roles, chronic exposure to either pain or stress can lead to maladaptive hormonal and neuronal modulations that can result in chronic pain and a wide spectrum of stress-related disorders including anxiety and depression. By inducing allostatic changes in the mesolimbic dopaminergic pathway, both chronic pain and stress disorders affect the rewarding values of both natural reinforcers, such as food or social interaction, and drugs of abuse. Despite opioids representing the best therapeutic strategy in pain conditions, they are often misused as a result of these allostatic changes induced by chronic pain and stress. The kappa opioid receptor (KOR) system is critically involved in these neuronal adaptations in part through its control of dopamine release in the nucleus accumbens. Therefore, it is likely that changes in the kappa opioid system following chronic exposure to pain and stress play a key role in increasing the misuse liability observed in pain patients treated with opioids. In this review, we will discuss how chronic pain and stress-induced pathologies can affect mesolimbic dopaminergic transmission, leading to increased abuse liability. We will also assess how the kappa opioid system may underlie these pathological changes.

## Introduction

In this mini review we will summarize the current understanding of mesolimbic dopamine signaling adaptations in response to chronic pain and stress and how these modifications can lead to opioid misuse liability. The dynorphin/kappa opioid receptor (KOR) system is highly involved in both stress and chronic pain processing. Therefore, it is likely that a shared mechanism drives these two negative affective states, which in turn alters rewarding/reinforcing properties. Here we will discuss how pain and stress decrease reinforcer-induced dopaminergic release in the nucleus accumbens (NAc), the role of dynorphin/kappa system in these pain/stress-induced alterations in dopaminergic transmission and how this may contribute to opioid abuse in pain patients.

## Pain and stress dysregulate the mesolimbic reward pathway

Pain and stress have a primary protective role that is critical for survival. That said, these states often lead to a drastic decrease in quality of life when their presence becomes maladaptive, such as in chronic pain and stress disorders. The transition from protective to pathological states is likely due to the allostatic nature of pain and stress. Allostasis enables a physiological system to maintain stability when exposed to stimuli that induce changes outside the normal homeostatic range (Koob and Le Moal, 2001; McEwen and Wingfield, 2003). However, during prolonged exposure to such stimuli, maintaining physiological stability can lead to maladaptive, often permanent changes that can manifest as stress disorders and chronic pain (Narita et al., 2004; Wang et al., 2011) (for more detail see reviews Elman et al., 2013; Elman and Borsook, 2016).

Growing evidence has implicated the mesolimbic pathway in the regulation of stress disorders, such as depression and anxiety (Nestler and Carlezon, 2006; Elman et al., 2009; Russo and Nestler, 2013; Polter and Kauer, 2014), as well as in pain sensation (Baliki et al., 2010), anticipation of analgesia or placebo-induced analgesia (Scott et al., 2008; Tracey, 2010) and chronic pain (Elvemo et al., 2015; Martikainen et al., 2015). The mesolimbic pathway is part of the principle reward-mediating system in the mammalian brain, which is composed of neurons projecting reciprocally from the ventral tegmental area (VTA) of the midbrain to the nucleus accumbens (NAc) in the forebrain. The dopaminergic neurons emerging from the VTA release dopamine in the NAc during reinforcers, such as food, social interaction or drugs of abuse. The NAc, in part through this dopaminergic transmission, plays a central role in mood-related and motivated behavior. It plays an important role in encoding salience, integrating reinforcing and aversive values of stimuli, and the motivation to seek or avoid these stimuli (O'Doherty, 2004; Montague et al., 2006; Schulz, 2006).

Interestingly, clinical studies link chronic pain conditions to aberrant functioning of the circuits involved in mood and motivation, including the mesolimbic pathway (Oluigbo et al., 2012; Baliki and Apkarian, 2015). Different subsets of neurons in the VTA can either be activated or inhibited by painful stimuli, such as a noxious thermal stimulus, tail pinch or footshock (Becerra et al., 2001; Ungless et al., 2004; Brischoux et al., 2009; Budygin et al., 2012). This heterogeneous response of the VTA to painful stimuli is also observed in the NAc. Indeed, dopamine release can be decreased (Leitl et al., 2014a), unchanged (Navratilova et al., 2012; Xie et al., 2014) or increased (Becerra et al., 2001; Becerra and Borsook, 2008; Baliki et al., 2010) depending on the type of pain and choice of pain paradigm. Studies using predictable pain stimuli show increased NAc activation that is likely induced by predictive noxious cues (Baliki et al., 2010; Becerra et al., 2001; Becerra and Borsook, 2008). Despite clear evidence of distinct NAc subregions (Thompson and Swanson, 2010; Castro and Berridge, 2014; Al-Hasani et al., 2015), discrimination between subregions of the NAc has not been directly investigated and compared in these studies. However, it is clear that the relief of ongoing pain and/or termination of a painful state increases dopamine transmission leading to negative reinforcement behaviors (Kalivas and Duffy, 1995; Seymour et al., 2005; Budygin et al., 2012; Navratilova et al., 2012; Xie et al., 2014). In summary, the dysregulation of dopaminergic transmission in the presence of chronic pain can lead to an imbalance in allostasis, which may underlie changes in the reinforcing properties of rewards. Indeed, patients experiencing chronic pain show reduced NAc activity and alterations in reward evaluation, decision making, and motivation tasks (Apkarian et al., 2004; Verdejo-García et al., 2009; Walteros et al., 2011). Furthermore, the termination of pain is negatively associated with NAc activity in chronic pain patients (Baliki et al., 2010) suggesting a dysregulation of dopaminergic function. These allostatic changes can alter the encoding of reinforcing values of further rewards, which in turn may lead to drug misuse.

Much research has been devoted to the interactions between stress and drug intake/reward. These studies have clearly identified interactions between stress, glucocorticoids and mesolimbic dopaminergic neurons, which all drive vulnerability to drugs of abuse. Stress, like drugs of abuse, activates the mesolimbic pathway. Exposure to acute stress, such as restraint and shock (Copeland et al., 2005; Ling et al., 2009) have been shown to induce dopamine release in the NAc (Thierry et al., 1976; Herman et al., 1982; Abercrombie et al., 1989; Kalivas and Duffy, 1995). Substance P and endogenous opioids, through the activation of dopaminergic neurons in the VTA, underlie this stress-induced dopamine release (Bannon et al., 1983; Kalivas and Abhold, 1987). Recent evidence shows that the stress-induced dopamine release in the NAc is inhibited when corticotrophin releasing hormone (CRH) receptor antagonists are injected into the VTA (Holly et al., 2015), confirming the involvement of stress-induced hormones in the control of dopamine efflux from the VTA to the NAc. Importantly, the hypothalamic-pituitary-adrenal (HPA) axis and glucocorticoid system indirectly alter dopamine transmission through enhancement of glutamate activity in the VTA (Härfstrand et al., 1986). Furthermore, glucocorticoids modulate the transmission of other stress-related neuropeptides, such as dynorphin, enkephalin, tachykinin, and neurotensin, particularly in the basal ganglia and nucleus accumbens (Chao and McEwen, 1990; Ahima et al., 1992; Schoffelmeer et al., 1996); for review, see (Angulo and McEwen, 1994). Conversely, chronic stress decreases dopamine transmission in the NAc (Quintero et al., 2000; da Silva Torres et al., 2003; Wood, 2004). These changes in dopaminergic tone following chronic stress are further confirmed by a reduction in the number of DAT bindings sites (Scheggi et al., 2002). It has also been shown that chronic drug exposure engages brain stress systems (Koob, 2013) such as noradrenaline, adrenocorticotrophic hormone, corticosterone, and CRH (Delfs et al., 2000; Koob, 2013). These different stress systems converge in the VTA to modulate its neuronal activity and consequently dopamine release regulation in the NAc. Activation of brain reward systems with concomitant activation of the HPA axis ultimately increases activity of brain stress systems. These actions may contribute to abuse potential through a negative affective state that increases over time and with repeated administration of drugs.

The distinction between how acute and chronic stress regulate dopamine transmission is particularly important in pain perception. Synaptic changes in VTA dopaminergic neurons occur in chronic stress conditions and these modifications may underlie allostatic adaptations to the persistence of pain. Such changes may induce or perpetuate stress-related disorders, such as anxiety and depression (da Silva Torres et al., 2003; Wood, 2004). It has been shown that prolonged exposure to stress results in hyperalgesia and it is postulated that this is due to the attenuation in dopaminergic activity in the NAc (Quintero et al., 2000; da Silva Torres et al., 2003; Wood, 2004).

Thus, far we have highlighted the critical role of the mesolimbic dopaminergic pathway in both pain and stress behaviors, which potentially contribute to changes in the reinforcing properties of drugs or natural rewards. In the following section we will discuss the role of the dynorphin/ KOR system in the regulation of the mesolimbic pathway during these pathological states.

## Dopamine and kappa opioid system in chronic pain and stress disorders

It is well documented that positive reinforcement is decreased in the presence of chronic pain (Shippenberg et al., 1988; Martin et al., 2004; Cahill et al., 2013; Leitl et al., 2014a,b; Hipólito et al., 2015). This chronic pain-induced alteration has been linked to a decrease in reinforcer-induced dopaminergic transmission (Niikura et al., 2010; Loggia et al., 2014; Hipólito et al., 2015; McDougle et al., 2015). Despite this evidence only few studies have assessed the impact of pain on opioid intake in preclinical studies. Most studies have used a conditioned place paradigm to test the reinforcing properties of opioids in rodents undergoing neuropathic or chronic pain (Ozaki et al., 2002; Narita et al., 2005; Cahill et al., 2013; Taylor et al., 2015). Interestingly, Wu et al. (2014) revealed that the known reinforcing doses of morphine were unable to induce a place preference under painful conditions. However, animals exposed to chronic pain developed a clear preference for the morphine-paired side when the dose of morphine was increased (Wu et al., 2014). In line with these findings, rodents self-administering opioids while experiencing pain show a decrease in their drug consumption of low doses, compared to controls (Lyness et al., 1989; Martin and Ewan, 2008; Wade et al., 2013; Hipólito et al., 2015; Taylor et al., 2015). This opioid consumption was, however, increased when high doses were accessible (Hipólito et al., 2015). Together these important results suggest a rightward-shift in the dose-response for opioid consumption in conditions of chronic pain. Importantly, this correlates with modifications in dopaminergic transmission from the VTA to the NAc (Hipólito et al., 2015). Dopaminergic release in the NAc is highly controlled by the opioid system, and Hipólito et al. (2015), demonstrated that pain induces a desensitization of mu-opioid receptors in the VTA during inflammatory pain (Hipólito et al., 2015). These changes in opioid receptor function led to decreased heroin- and DAMGO-induced dopamine release in the NAc.

**Figure 1:**
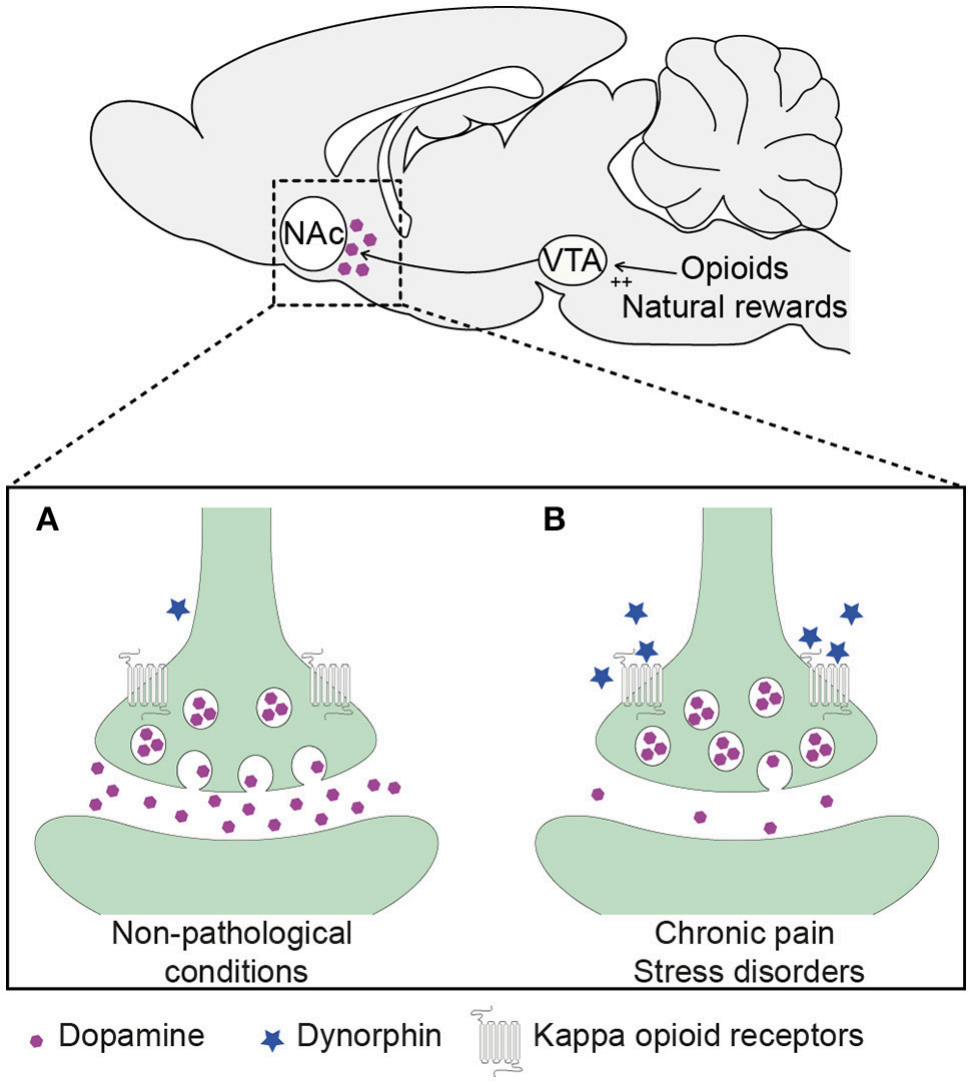
**Schematic representation of dynorphin/kappa opioid receptor system regulation on dopamine release in the nucleus accumbens in (A) physiological conditions and (B) chronic pain and stress disorders conditions**.

The KOR system, may also be involved in these changes in dopamine release. Evidence points toward a role for the KOR system in many of the changes induced by chronic pain (Cahill et al., 2014). The dynorphin/KOR system is composed of prodynorphin peptides and KOR, a seven-transmembrane spanning Gi/o protein-coupled receptor (GPCR) are expressed throughout the brain (Le Merrer et al., 2009; Cahill et al., 2014). KOR are expressed on presynaptic terminals of dopaminergic neurons in the NAc (Werling et al., 1988; Ebner et al., 2010; Al-Hasani and Bruchas, 2011). Activation of these receptors decreases dopamine release (Spanagel et al., 1992; Margolis et al., 2003) (Figure 1), a phenomenon known to modulate aversive and negative emotional states (Wadenberg, 2003; Cahill et al., 2014; Wise and Koob, 2014).

In conjunction with the data showing that inflammatory pain decreases morphine- and heroin-induced NAc dopamine release and impairs the rewarding effects of morphine (Narita et al., 2005; Hipólito et al., 2015), Narita et al. (2005) showed that pain-induced attenuation in place preference can be reversed by systemic or local NAc blockade of KOR using norbinaltorphimine (NorBNI), a highly selective antagonist for KOR. The aversive component of exogenous KOR stimulation, measured by place preference conditioning, is also suppressed when animals are experiencing inflammatory pain conditions (Shippenberg et al., 1988) suggesting the presence of a kappa opioid tone during painful conditions that induces a sustained dysphoric state (Figure 1).

There is, however, some controversy regarding the role of the dynorphin/kappa opioid system in regulating the reinforcing properties of rewards during pain. Some studies showed that KOR antagonism during pain did not reverse the pain-induced decrease in intracranial self-stimulation of mesolimbic pathway in rats (Leitl et al., 2014a,b). These discrepancies could be explained by the presence of hot and cold spots, two distinct areas in the NAc shell in which activation of KOR can drive either aversive or reinforcing behaviors (Castro and Berridge, 2014; Al-Hasani et al., 2015). Systemic application of KOR antagonists likely targets both of these discrete areas while microinjections of KOR agonists/antagonists specifically target discrete areas within the NAc, yielding opposing behaviors and potential interpretations.

Dynorphin is considered a mediator of dysphoria-like behavior and, as a result, a primary mediator of the anti-reward effects that occur during drug withdrawal, drug craving, and relapse to drug seeking. During stress, dynorphin activates KOR on dopaminergic cell bodies and terminals to decrease striatal dopamine levels and inhibit the firing of dopamine neurons. In particular, several reports demonstrate that KOR agonists inhibit dopamine release specifically via action within the NAc (Shippenberg et al., 2007; Ebner et al., 2010; Graziane et al., 2013; Tejeda et al., 2013). These stress-induced cellular alterations in turn produce reinstatement, aversion, and negative affective-like behavioral states (Land et al., 2008; Bruchas et al., 2011; Graziane et al., 2013; Tejeda et al., 2013; Van't Veer and Carlezon, 2013). Chronic stress leads to prolonged activation of KORs, which may contribute to reduced dopamine function, and is correlated with negative affective states (Koob, 2013). Furthermore, stress is known to increase drug and alcohol self-administration, and KOR activation potentiates this effect. Blocking KOR, however, inhibits stress-induced escalations in cocaine self-administration, alcohol intake, and preference for nicotine (Redila and Chavkin, 2008; Sperling et al., 2010). Prolonged activation of KORs causes a significant elevation in ICSS thresholds, a behavior indicative of anhedonia (Chartoff et al., 2016). It is this anhedonia and negative affective state that motivates increased drug taking (Baarendse and Vanderschuren, 2012).

In order to understand these mechanisms, recent neural circuitry studies have focused on projections into the NAc, and how these may be involved in the modulation of dopamine during aversive behaviors. Specifically, projections from the basolateral amygdala (BLA) to the NAc have been shown to modulate dopamine-mediated neuronal responses to both stress and opioid reward salience (Lintas et al., 2011, 2012; Stuber et al., 2011; Namburi et al., 2015). Interestingly, opioid exposure and withdrawal are associated with marked hypofunction of dopaminergic transmission (Diana et al., 1995), which may ultimately drive the need for higher doses of opioid analgesics. Stress-induced changes in plasticity have been extensively studied for mood disorders, but these adaptations are poorly understood in the context of chronic pain, despite evidence that clearly suggests that stress plays a key role in exacerbating pain symptomology.

Though this review has primarily focused on the VTA and the NAc, it is important to acknowledge the role of other brain regions critical in the regulation of pain, stress and reward responses. The amygdala is very much involved in the processing of both positive and negative valence (see review Janak and Tye, 2015). Specifically the BLA and the central nucleus of the amygdala play important roles in affective pain in addition to better studied roles in the processing of mood and fear disorders and reinforcement (Pare and Duvarci, 2012; Veinante et al., 2013). More recently it has been shown that habenula to NAc dopaminergic neurons drive inhibitory anti-reward tone during stress and pain conditions (Lee and Goto, 2011). The lateral hypothalamus, a region critical to positive reinforcement also plays a role in the pain response through sensory mechanisms (Ezzatpanah et al., 2015). These structures also contribute to increases in norepinephrine, CRH, vasopressin, hypocretin, and substance P, driving a stress-like emotional state.

## Future directions

The alterations in the dopaminergic system induced by either pain or stress can generate long-term modifications in the reinforcing values of opioids and thus lead to misuse. Therefore, it is important to elucidate how these modifications are manifested at the cellular level in the mesolimbic pathway. To date, few studies have assessed the impact of pain and stress together on opioid intake in rodent models. One critical factor that is particularly pertinent when studying chronic pain-induced disorders is experimental/sampling time. Many preclinical models used previously were deemed as failures (Yalcin and Barrot, 2014), but this may have been simply due to timing. For example many of the same studies carried out during the first 3 weeks of pain induction vs. after the first 3 weeks show strikingly opposite results (see review Yalcin and Barrot, 2014).

In addition to the importance of improving models of chronic pain and stress to assess their involvement in misuse liability, a deeper understanding on the intricate details of neuromodulation and signaling within key brain structures is critical. Recently, two studies revealed that KOR activation in discrete regions of the NAc is not only anhedonic and aversive but can also be reinforcing (Castro and Berridge, 2014; Al-Hasani et al., 2015). Remarkably, these studies revealed the presence of both a hedonic and anhedonic KOR areas in the NAc in both mice and rats (Castro and Berridge, 2014; Al-Hasani et al., 2015). These findings further enhance the complexity of the KOR system in regulating the rewarding and aversive components of external stimuli and demands further study for how these newly identified systems modulate the pain experience.

There is clear co-morbidity between chronic pain and stress-induced pathologies. Concomitant dysregulation of mesolimbic dopaminergic transmission is thought to increase opioid abuse vulnerability. To reduce the abuse potential of opioid analgesics a better understanding of the interactions between pain and stress systems is required. In these efforts, stress-related systems, such as the kappa opioid system have been identified as a key system in the regulation of dopamine release during pain and stress (Figure 1). This system may be crucially involved in driving the pathological changes that result in misuse and potential fatalities.

## Author contributions

NM, RA wrote the manuscript. NM, JM, RA conceived of the concept and structure of the manuscript.

### Conflict of interest statement

The authors declare that the research was conducted in the absence of any commercial or financial relationships that could be construed as a potential conflict of interest.
